# Vulnerable Older Adults in Disasters: Effects of Hurricane Irma on Nursing Homes and Assisted Living

**DOI:** 10.1093/geroni/igaa057.2607

**Published:** 2020-12-16

**Authors:** Lindsay Peterson, David Dosa, Patricia D’Antonio

## Abstract

Preparedness of residents in long-term care (LTC) in the face of hurricane emergencies is a contested and largely unanswered question. Our prior work involving the U.S. Gulf Coast hurricanes of 2005-08 showed that exposure to various storms on nursing home (NH) residents resulted in significantly more deaths than reported by health care officials. This work also highlighted that evacuation of NH residents, compared to sheltering in place, was independently associated with morbidity and mortality. Hurricane Irma struck Florida on Sept. 10, 2017, prompting the evacuation of thousands of NH and assisted living community (ALC) residents. This symposium will discuss the effects of Hurricane Irma on vulnerable older adults residing in NHs and ALCs using mixed quantitative and qualitative methodologies. The first presentation will discuss morbidity and mortality of NH residents exposed to Hurricane Irma and will stratify by long stay/short stay status and hospice enrollment. The second presentation will discuss improvements and continued barriers to NH preparedness based on interviews with 30 administrators following Hurricane Irma. Using a novel methodology to identify residents of ALCs using secondary data sources, the third presentation will document AL resident morbidity and mortality risk following Hurricane Irma. The final presentation will highlight results of interviews with 70 stakeholders from small and large ALCs concerning the hurricane experiences of residents, including those with dementia. This symposium offers a multi-faceted view of a disaster’s effects on LTC residents across Florida, including novel data from the NH environment and lesser-examined ALCs.

